# Physiological characterization of *Kluyveromyces marxianus* strains for the development of a microbial platform to obtain bioethanol from cheese whey permeate

**DOI:** 10.3389/fmicb.2025.1663736

**Published:** 2025-10-01

**Authors:** Claudia De Micco, Matteo Radice, Nicola Di Fidio, Gennaro Agrimi, Isabella Pisano

**Affiliations:** ^1^Department of Bioscience, Biotechnology and Environment, University of Bari “Aldo Moro”, Bari, Italy; ^2^Faculty of Earth Sciences, Departamento de Ciencia de La Tierra, Universidad Estatal Amazónica, Puyo, Ecuador; ^3^Interuniversity Consortium for Biotechnology (CIB), Trieste, Italy

**Keywords:** *Kluyveromyces marxianus*, cheese whey permeate, ethanol, β-galactosidase, fermentation

## Abstract

The increasing interest in renewable feedstocks for biochemicals production includes the valorization of cheese whey (CW), a by-product of the dairy industry. CW contains a high concentration of lactose, accounting for most of its organic load, making it a major environmental pollutant if untreated. A valorization approach of CW is based on the separation of valuable proteins by ultrafiltration, and the obtainment of a lactose-rich cheese whey permeate (CWP). The conversion of lactose in CWP into bioethanol is considered a sustainable solution for the valorization of this waste. However, lactose is not metabolized by the yeast species *Saccharomyces cerevisiae*, namely the most industrially used microorganism, to produce bioethanol. Differently, the non-conventional yeast *Kluyveromyces marxianus* shows high growth rates on a broad range of industrially relevant substrates, including lactose. This study provides a comprehensive physiological characterization of three *K. marxianus* strains (DSM 5422, DSM 7239, and DSM 5572) to evaluate their potential for lactose hydrolysis and fermentation in order to produce advanced bioethanol from CWP. Microplate growth tests, β-galactosidase activity assays, and flask fermentations were carried out to identify optimal strains for efficient CWP valorization, advancing the applications of *K. marxianus* in industrial biotechnology. The optimal conditions for lactose utilization and ethanol production were identified as 130 g/L of lactose at 42 °C in semi-synthetic media (SSM). Even though strain DSM 7239 showed the highest β-galactosidase activity of 27.8 ± 0.9 U mg^−1^, strains DSM 5422 and 5572 were identified as the best performing strains at shake flask experiments in terms of ethanol yield on the substrate, with 0.48 ± 0.03 g/g and 0.50 ± 0.03 g/g, respectively, after 1 day of fermentation.

## 1 Introduction

The recovery and sustainable exploitation of industrial waste is a theme that cuts across all production chains, and there are international policies that introduce guidelines to solve this problem. An example is represented by goal 12 of the United Nations 2030 Agenda, where “Ensure sustainable consumption and production patterns” is identified among the priorities (Tsalis et al., 2020). Cheese whey (CW), the liquid by-product resulting from the curdling process in cheese-making (Osorio-González et al., 2022), is considered one of the most important pollutants of the dairy industry due to the presence of lactose and hardly-biodegradable proteins. Furthermore, several studies estimated a growing production of CW in the next decades (Lappa et al., 2019; Figueroa Pires et al., 2021). This industrial waste represents approximately 90% of the milk volume (Figueroa Pires et al., 2021) and retains 55% of the milk nutrients (Pendón et al., 2021). Specifically, most of the lactose in milk remains in the CW, contributing to 90% of the total organic load; with the remaining part represented by fats and proteins (Prazeres et al., 2012; Figueroa Pires et al., 2021). CW is characterized by high Biochemical Oxygen Demand (BOD) and Chemical Oxygen Demand (COD) values, equal to 27–60 kg m^−3^ and 50–102 kg m^−3^, respectively (Prazeres et al., 2012; Rocha and Guerra, 2020). The growing interest in the use of renewable feedstock to produce biochemicals, biomaterials, and biofuels also involves agro-industrial and food waste, such as CW. Significant financial resources are needed for CW disposal in order to lower the polluting load and prevent environmental issues like excessive oxygen consumption, soil eutrophication and depletion, and groundwater contamination (Caballero et al., 2021; Dinkci, 2021). Because of the high nutritional content, roughly 50% of whey is utilized for food (Asunis et al., 2020; Zotta et al., 2020; Barba, 2021) and animal nutrition sectors (El-Tanboly et al., 2017; Palmieri et al., 2017; Zou and Chang, 2022; Arshad et al., 2023).

A biorefinery approach for the valorization of this dairy by-product is the extraction of valuable proteins by membrane filtration methods and the recovery of a lactose-rich effluent called cheese whey permeate (CWP) (Nobre Macedo et al., 2020; Zandona et al., 2021). Nonetheless, a key challenge is that many wild-type microorganisms are unable to metabolize lactose directly. Over the past two decades, various methods have been developed to enable *Saccharomyces cerevisiae* to consume lactose, including enzymatic conversion of lactose into glucose and galactose using free or immobilized enzymes, as well as genetic modifications to enhance lactose metabolism (Zou and Chang, 2022). However, most strains showed unwanted characteristics, such as genetic instability or inability to produce ethanol with high concentration and/or yield (Rubio-Texeira, 2006; Zou et al., 2013).

Since the purchase of the commercial β-galactosidase enzyme at the industrial process scale is not compatible with a cost-effective process, non-conventional yeast strains that naturally exhibit β-galactosidase activity, such as *Kluyveromyces marxianus*, have been recently studied (Rocha and Guerra, 2020). *K. marxianus* exhibits superior traits, such as the ability to grow on a wide range of substrates with high specific growth rates, tolerance to high temperatures (up to 52 °C) (Fonseca et al., 2008), as well as the capacity to produce ethanol with high yield and productivity and valuable bioproducts, such as proteins and cell biomass (Carvalho et al., 2021). Together with the GRAS (Generally Recognized As Safe) classification, these features make this yeast an appealing microorganism for application in CW valorisation (Martynova et al., 2016; Rouanet et al., 2020).

Up to now, the majority of the biotechnological processes reported in the scientific literature, aiming to convert lactose present in CW, is based on the use of β-galactosidase for the preliminary hydrolysis of lactose to give its two monosaccharides, namely glucose and galactose (Awasthi et al., 2022). Lactose hydrolysis has various advantages, including nutritional and technical benefits; indeed, due to the increasing lactose-intolerant population, the commercial interest in β-galactosidase manufacturing is tied to large-scale food and medicinal uses (Fonseca et al., 2008; Rollini et al., 2008). As well documented in the literature, *K. marxianus* can be effectively used for the production of β-galactosidase from CW (Bansal et al., 2008; Yadav et al., 2014; Bilal et al., 2022; Singh and Sambyal, 2023). Moreover, only a few *Kluyveromyces* yeast species have significant potential for the efficient production of bioethanol from CWP (Díez-Antolínez et al., 2018). Particularly, *K. marxianus* exhibits advantageous physiological traits, such as the ability to thrive in harsh industrial environments and effectively utilize lactose-rich substrates, that make it a promising whole-cell biocatalyst for simultaneous production of bioethanol and β-galactosidase starting from industrial side-streams and/or waste in the perspective of modern circular bioeconomy (Beniwal et al., 2017; Karim et al., 2020).

However, from an industrial point of view, the selection of a proper yeast strain with appropriate physiological features is crucial to ensure an efficient lactose utilization from CWP and a competitive and selective production of ethanol and β-galactosidase with respect to other yeast species (Fonseca et al., 2013; Morrissey et al., 2015).

In this context, the present study aims to provide a comprehensive physiological characterization of three *K. marxianus* strains and assess their potential for the sustainable and profitable valorization of CWP, mainly through bioethanol production. In particular, *K. marxianus* DSM 5422, *K. marxianus* DSM 7239, and *K. marxianus* DSM 5572 were selected due to their ability to efficiently ferment lactose (Ozmihci and Kargi, 2007a,b,c,d; Lane et al., 2011; Rocha et al., 2011; Ortiz-Merino et al., 2018; Leandro et al., 2019; de Albuquerque et al., 2021). The proposed physiological characterization was mainly focused on the study of the β-galactosidase enzyme activity for lactose hydrolysis and its correlation to the fermentation of CWP to ethanol. For this purpose, preliminary microplate growth tests on various carbon sources and measurements of β-galactosidase enzyme activity were carried out to increase the knowledge of *K. marxianus* physiology and increase its industrial biotechnology applications (Fonseca et al., 2008). Subsequently, flask fermentation tests were carried out to evaluate the performance of these three strategic yeast strains in ethanol production from the waste-derived CWP and define the best microorganism for the proposed third-generation biorefinery model.

## 2 Materials and methods

### 2.1 Strains and maintenance

Three wild-type *K. marxianus* strains, obtained from the Deutsche Sammlung von Mikroorganismen und Zellkulturen (DSMZ), were used in this study: *K. marxianus* DSM 5422; *K. marxianus* DSM 7239, *K. marxianus* DSM 5572. The lyophilized samples were reactivated by an overnight (ON) pre-cultivation in YPD liquid medium containing 10 g/L yeast extract, 20 g/L peptone and 20 g/L D-glucose, at 30 °C in a rotary shaker incubator at 200 rpm. The pre-cultures were inoculated in 4 mL of YPD (30 °C, 200 rpm, ON). From each second pre-cultivation, 1 mL stock was obtained for each strain to which 15% glycerol (v/v) was added and then stored at −80°C for cell banking. One Petri dish of YPD agar (YPDA) (10 g/L yeast extract, 20 g/L peptone, 20 g/L D-glucose and 15 g/L agar) was plated from each tube, plus one control to check the sterility. After 48 h of incubation at 30 °C, they were stored at 4 °C. Strains were maintained on YPDA and sub-cultured monthly.

### 2.2 Inoculum preparation

A pre-culture for each experiment was inoculated from a single colony plated on YPDA. Sterile tubes containing YPD medium were inoculated, one tube for each strain plus one negative control for sterility. Unless otherwise specified, the standard growth condition was 4 mL of YPD in 15 mL sterile tubes. Pre-cultivations were incubated at 30 °C in a rotary shaker at 200 rpm. To determine growth kinetics or conduct the β-galactosidase test, after an ON pre-culture, the optical density was measured at 600 nm (OD_600_), and cells were inoculated in the appropriate media, with an initial OD_600_ of 0.1. Before the inoculum, cells were washed at least twice with sterile demineralized water (dH_2_O). This involved two subsequent steps of centrifugation (5 min at 3,600 RCF) and resuspension in an appropriate amount of sterile dH_2_O.

### 2.3 Preparation of culture media

The semi-synthetic medium (SSM) was adapted from literature (Martynova et al., 2016) and contained 5 g/L yeast extract, 0.7 g/L MgSO_4_, 1 g/L KH_2_PO_4_, 0.1 g/L K_2_HPO_4_, and 5 g/L (NH_4_)_2_SO_4_ (pH 5.5). After autoclaving (120 °C, 20 min), the medium was cooled to room temperature. Lactose was prepared as a 300 g/L stock solution and autoclaved separately, then added to the medium in the appropriate ratio based on the experimental conditions. CWP was gently provided by the Italian company Distilleria Bartin Srl. CWP contained 140 g/L of lactose, 1 g/L of total nitrogen, 4,000 ppm of mineral salts and was characterized by a pH of 5 ± 0.5. The protein content of 6.3 g/L was determined by the Bradford assay (Bradford, 1976). After the collection, CWP was stored at 4 °C for no more than 24 h. CWP was then autoclaved in a sealed 1 L Schott bottle at 121 °C for 15 min. Precipitated solids were allowed to settle ON before the upper phase, rich in lactose, was used for fermentations (Löser et al., 2015). Sterile whey was kept at 4 °C and used as medium.

### 2.4 Growth curves in microtiter plates

Yeast cultures were pre-grown in tubes as described for the inoculum preparation. After an ON incubation (30 °C, 200 rpm), the OD_600_ was measured to calculate the proper volume of the inoculum suitable to reach an initial OD_600_ of 0.1. Three different carbon sources were tested for each strain: lactose, glucose, and galactose. Each carbon source was prepared as a stock solution of 300 g/L, sterilized at 121 °C for 15 min and then mixed in the proper proportion with the SSM medium to obtain the final concentrations of sugar equal to 50 and 2 g/L. Growth tests were conducted in a 96-well microtiter plate (MTP) system using the Sunrise^TM^ absorbance microplate reader (Tecan). Sterile round-bottom microtiter plates were used, covered with a transparent sealing film permeable to gases to ensure sterility. The working volume was 350 μL, while the starting OD_600_ was 0.1. A well containing only the medium was prepared to correct the respective absorbance at each time point. For each condition, yeast growth was monitored for 15 h. Data was collected using the software Magellan^TM^. The maximum specific growth rate μ_max_ (h^−1^) was calculated during the exponential growth phase to compare the growth performances of the three strains, according to the literature (Rocha et al., 2011). Each fermentation condition was performed in triplicate. Moreover, the results shown are the average of at least two independent experiments.

### 2.5 β-galactosidase tests

#### 2.5.1 β-galactosidase enzyme assay

*K. marxianus* cultures were pre-grown in 15 mL tubes in YPD medium as described for the inoculum preparation (Section 2.2). After an ON incubation (30 °C, 200 rpm), the OD_600_ was measured to calculate the proper volume to be used for the inoculum. Then, cultures were inoculated in the SSM containing 50 g/L lactose with an OD_600_ equal to 0.1 and incubated at 30 °C and 200 rpm. Since lactose induces the expression of β-galactosidases, cultures were grown in a lactose-containing medium (Liu et al., 2022). Unless otherwise specified, the standard growth condition was 5 mL of SSM-lactose 50 g/L in 15 mL sterile tubes. At specific times, samples were collected to conduct the β-galactosidase activity test. Cells were pelleted for 4 min at 1,520 RCF, resuspended in 565 μL of sterile PBS and permeabilized with sodium dodecyl sulfate (SDS)/chloroform treatment for enzyme extraction, according to the literature (Panesar et al., 2006; Bansal et al., 2008; de Faria et al., 2013; Karim et al., 2020). For the permeabilization step, 15 μL of chloroform and 20 μL of 2 g/L SDS solution were added to each reaction tube. The samples were vortexed and incubated for 5 min at 30 °C. The reaction was started by adding 115 μL of *o*-nitrophenyl-β-galactose (ONPG) 1.25 mM. Finally, the reaction was stopped after 10 min by adding 285 μL of Na_2_CO_3_ 1.0 M, and the absorbance was read at 420 nm to measure the extracellular enzyme activity. ONPG and SDS solutions were prepared fresh by dissolving the powders in sterile PBS. To calculate the β-galactosidase specific activity, the *o*-nitrophenol (ONP) concentration was first determined by using the Beer-Lambert law. In fact, when the β-galactosidase cleaves the chromogenic substrate ONPG, ONP is released, producing an orange-yellowish color which is proportional to the amount of ONP concentration. The molar extinction coefficient of ONP at 30 °C was 4,500 M^−1^ cm^−1^, according to the literature (Sheetz and Dickson, 1980; Inchaurrondo et al., 1994; Becerra et al., 1998). One enzyme unit (U) was defined as the amount of enzyme that hydrolyzed 1 μmol of substrate per minute under the stated conditions. Specific activity was expressed as U per mg of biomass and in U per mL for the activity calculated on the cell's supernatant. The results presented were the average of three independent experiments.

#### 2.5.2 Kinetics of o-nitrophenyl-β-galactose hydrolysis

Cultures were pre-grown in 15 mL tubes in YPD medium as previously described. After 12 h, the OD_600_ was measured to calculate the inoculum volume. Then, cultures were inoculated in 5 mL of SSM-lactose 50 g/L medium in 15 mL sterile tubes with an initial OD_600_ 0.1 at 30 °C and 200 rpm. After ON growth, the OD_600_ was measured by using the Tecan Sunrise^TM^ MTP reader to calculate the volume of cells needed to start the test with a higher cell concentration equal to OD_600_ 0.5. As before mentioned, cells were permeabilized with SDS/chloroform treatment. The protocol in the MTP was set according to the literature (Griffith and Wolf, 2002). Briefly, after withdrawing the volume of culture according to the number of samples to run, cultures were centrifuged for 4 min at 1,520 RCF. The supernatant was removed, and cells were resuspended in 250 μL of PBS. The permeabilization was carried out as previously described. After this step, 250 μL of the sample was aliquoted in a 96-well plate, adding 50 μL of ONPG at increasing concentration and 125 μL of PBS to obtain a final working volume of 425 μL. A wide shake mode speed was set (14.2 mm, 2 Hz). Absorbance kinetic data at 405 nm were collected every 30 s. Kinetic measurements at six increasing ONPG concentrations (1.7, 3.4, 6.8, 12.5, 25.0, and 50.0 mM) were performed in order to evaluate the different reaction rates (V_0_) at varying ONPG concentrations for the three yeast strains. The specific activity was expressed as V_0_ (μmol min^−1^) per mg of biomass. The analysis was carried out at 30 and 37 °C. A Lineweaver–Burk (1/V vs. 1/S) plot was used to determine the Michaelis-Menten parameters K_M_ (mM) and V_max_ (μmol min^−1^mg^−1^) when different concentrations of ONPG were used as the substrate. The parameters were calculated by a simple weighted non-linear regression of the Lineweaver–Burk plot. In particular, K_M_ and V_max_ values were calculated by using Equation 1, with a slope of K_M_/V_max_ and an intercept of 1/V_max_. Data represented the result of at least three independent experiments, with each measurement being the average of two technical replicates.


(1) $$\begin{array}{l} \frac{1}{V}=\frac{1}{V_{\max}}+\frac{K_{M}}{V_{\max}}\times \frac{1}{S} \end{array}$$

### 2.6 Flask growth tests

Fermentation tests were carried out at 30, 37, and 42 °C by using SSM supplemented with lactose or CWP as culture media. In flask fermentations, pre-cultures were obtained as previously described. After an ON incubation, cultures were washed twice in sterile water before the inoculum. Cultures were performed in 100 mL Erlenmeyer flasks with 40 mL of the appropriate media. Agitation speed was set at 200 rpm. During the process, culture samples were collected for analysis of OD_600_, pH, and subsequent extracellular concentration of the compounds. For this purpose, 1 mL of fermentation broth was harvested, centrifuged at 1,520 RCF for 5 min, and the supernatant was collected and stored at −20 °C for the following high-performance liquid chromatography (HPLC) analysis.

### 2.7 Analytical methods

#### 2.7.1 Determination of cell biomass

*K. marxianus* growth was quantified by measuring the optical density at 600 nm using a spectrophotometer. The cell mass was calculated from the OD_600_ value within the linear range of the OD_600_ vs. dry cell weight (DCW) for each strain. One OD_600_ unit corresponded to 0.665 ± 0.020 g/L of cell mass. For the final steady-state samples during flask experiments, DCW was directly measured by withdrawing 10 mL of culture from the flask, and the pellet was separated by centrifugation at 3,600 RCF for 5 min. The pellet was dried at 60 °C until a constant dry weight was reached. The specific growth rate was determined by linear regression of the plot ln OD_600_ unit vs. time (h), at the exponential growth phase.

#### 2.7.2 Determination of extracellular compounds concentration

The concentration of lactose and ethanol was determined by HPLC analysis using a Waters Alliance 2695 separation module equipped with a Rezex ROA-Organic Acid H^+^ (8%) 300 mm × 7.8 mm column (Phenomenex Inc., USA), coupled with a Waters 2410 refractive index detector and a Waters 2996 UV detector. The temperature of the oven was set at 60 °C, while the mobile phase was 2.5 mM H_2_SO_4_ with a flow rate of 0.5 mL/min. A calibration curve was obtained for each compound of interest by using external pure standards.

#### 2.7.3 Fermentation parameters calculation

The ethanol yield on the substrate (Y_P/S_) was calculated according to Equation 2 at different time points:


(2) $$\begin{array}{l} Y_{P/S}=\frac{\left(EtOH_{f}-EtOH_{i}\right)}{\left(Lac_{i}-Lac_{f}\right)} \end{array}$$

where EtOH_f_ and EtOH_i_ were the final and initial concentration of ethanol (in g/L), respectively, while Lac_f_ and Lac_i_ were the final and initial concentration of lactose (in g/L), respectively. According to the literature, the maximum theoretical ethanol yield from lactose is 0.538 g/g (Pasotti et al., 2017; Carvalho et al., 2021).

The biomass yield on the substrate (Y_X/S_) was calculated according to Equation 3 at the end of the fermentation:


(3) $$\begin{array}{l} Y_{X/S\;}=\;\frac{Biomass}{\left(Lac_{i}-Lac_{f}\right)} \end{array}$$

where the produced biomass was expressed in g/L.

### 2.8 Statistical methods

Unless otherwise stated, experiments were performed with three independent replicates. GraphPad Prism 10 software was used for statistical analyses. For the analysis of the specific enzyme activity at different times, temperatures and ONPG concentrations, a two-way ANOVA was used in combination with a Tukey's *post-hoc* test in order to perform multiple comparisons. The remaining statistical analyses were performed using a two-tailed, unpaired, Student's *t*-test. Statistical significance was established at *p* < 0.05 and marked by ^*^p < 0.05, ^**^p < 0.01, ^***^p < 0.001 and ^****^p < 0.0001. Alternatively, in the tables, values within the same column that share the same letters are not significantly different.

## 3 Results

### 3.1 Microtiter plate growth assays

The ability of the three selected *K. marxianus* strains (DSM 5422, DSM 7239, and DSM 5572) to grow on lactose and its hydrolysis products, namely glucose and galactose, was preliminarily investigated in micro-cultivation tests, as described in Table 1.

**Table 1:** Maximum specific growth rates (μ_max_), doubling time and maximum OD_600_ values in 96-well microtiter plates growth experiments of three *K. marxianus* wild-type strains on glucose, galactose, and lactose at two concentrations (20 and 50 g/L) and temperatures (30 and 37 °C).

***K. marxianus* strain**	**Carbon source (g/L)**	μ_**max**_ **(h**^**−1**^**)**		**Doubling time (h)**		**OD**_**600**_	
		**30** °**C**	**37** °**C**	**30** °**C**	**37** °**C**	**30** °**C**	**37** °**C**
DSM 5422	Glucose 20	0.34	0.37	2.03	1.89	1.26	1.44
	Glucose 50	0.30	0.41	2.33	1.72	1.34	1.59
	Galactose 20	0.24	0.29	2.87	2.38	0.97	1.02
	Galactose 50	0.22	0.31	3.18	2.23	1.08	1.25
	Lactose 20	0.27	0.36	2.57	1.93	1.30	1.42
	Lactose 50	0.26	0.41	2.63	1.72	1.35	1.53
DSM 7239	Glucose 20	0.32	0.44	2.20	1.59	1.48	1.49
	Glucose 50	0.36	0.37	1.91	1.90	1.53	1.58
	Galactose 20	0.23	0.27	3.08	2.55	1.45	1.47
	Galactose 50	0.25	0.25	2.83	2.83	1.47	1.54
	Lactose 20	0.26	0.32	2.70	2.16	1.41	1.54
	Lactose 50	0.30	0.31	2.36	2.22	1.57	1.58
DSM 5572	Glucose 20	0.32	0.33	2.17	2.11	1.19	1.27
	Glucose 50	0.30	0.34	2.30	2.02	1.38	1.47
	Galactose 20	0.24	0.27	2.87	2.55	1.19	1.25
	Galactose 50	0.25	0.28	2.83	2.53	1.23	1.36
	Lactose 20	0.26	0.32	2.69	2.16	1.19	1.31
	Lactose 50	0.26	0.32	2.64	2.18	1.28	1.60

The results were the mean values (n = 3), and the relative standard deviation values were < 15%.

According to the literature, various *K. marxianus* strains can be classified into efficient or scarce utilizers of lactose (Varela et al., 2017). On this basis, the growth test performed on glucose as the sole carbon source aimed at highlighting the differences in lactose metabolism. Galactose was also included as a carbon source in order to characterize strains eventually able to exploit all the sugars present in the CWP (Peri et al., 2024).

As reported in Table 1, the increase in temperature from 30 to 37 °C increased the μ_max_ values for all the strains grown on all the tested carbon sources at 20 and 50 g/L. Moreover, the μ_max_ values on glucose showed the highest values at both the tested temperatures, followed by lactose and galactose. The latter displayed the lowest values for all strains at both concentrations. The results obtained on galactose in the present study agreed with those reported by Beniwal et al. (2017) on all three strains at the flask scale.

*K. marxianus* DSM 5422 reported the highest μ_max_ values of 0.41 h^−1^ on 50 g/L glucose and lactose at 37 °C, *K. marxianus* DSM 7239 reported the highest μ_max_ value of 0.44 h^−1^ on 20 g/L glucose at 37 °C, while *K. marxianus* DSM 5422 showed its highest μ_max_ value of 0.34 h^−1^ on 50 g/L glucose at 37 °C. The final OD_600_ values achieved after 16 h of fermentation (Table 1) followed the same trend of μ_max_ values. The OD_600_ values achieved at 37 °C were higher than those obtained at 30 °C, suggesting that higher temperatures favor the metabolism toward more accelerated growth. *K. marxianus* DSM 7239 reached OD_600_ values higher than the other two strains, and comparable values were reached at 30 and 37 °C, while on 50 g/L lactose at 30 °C reached the highest value of OD_600_ compared to all other conditions tested among all strains at this temperature. Based on these results, glucose is a better substrate than lactose and galactose. Galactose was the carbon source that showed the slowest growth trend for all three screened strains, even though DSM 7239 reached an OD_600_ of about 1.5 after 16 h on 20 and 50 g/L galactose, but with a longer lag phase compared to the other two carbon sources.

In order to investigate the effect of lactose as the sole carbon source and simulate its high concentration in CWP (170–180 g L^−1^) (Díez-Antolínez et al., 2016), the growth ability of the three yeasts was tested on lactose concentrations ranging from 100 to 200 g/L at 30 and 37 °C (Table 2).

**Table 2:** Maximum specific growth rates (μ_max_), doubling time and maximum OD_600_ values in 96-well microtiter plates growth experiments of three *K. marxianus* strains on SSM at increasing lactose concentrations (100–200 g/L) at two temperatures (30 and 37 °C).

***K. marxianus* strain**	**Lactose concentration (g/L )**	μ_**max**_ **(h**^**−1**^**)**		**Doubling time (h)**		**OD**_**600**_	
		**30** °**C**	**37** °**C**	**30** °**C**	**37** °**C**	**30** °**C**	**37** °**C**
DSM 5422	100	0.24	0.37	2.85	1.82	1.17	1.50
	130	0.33	0.42	2.30	1.42	1.27	1.50
	160	0.33	0.40	2.34	1.30	1.13	1.38
	200	0.27	0.42	2.69	1.37	0.94	1.30
DSM 7239	100	0.30	0.32	2.30	2.12	1.48	1.46
	130	0.38	0.50	2.02	1.43	1.16	1.45
	160	0.36	0.42	2.17	1.48	1.04	1.26
	200	0.33	0.43	2.16	1.50	0.93	1.06
DSM 5572	100	0.27	0.34	2.55	2.01	1.24	1.49
	130	0.32	0.35	2.46	1.82	1.01	1.38
	160	0.30	0.33	2.55	1.70	0.96	1.39
	200	0.29	0.43	2.51	1.44	0.89	1.28

The results were the mean values (n = 3), and the relative standard deviation was < 15%.

**Figure 1:**
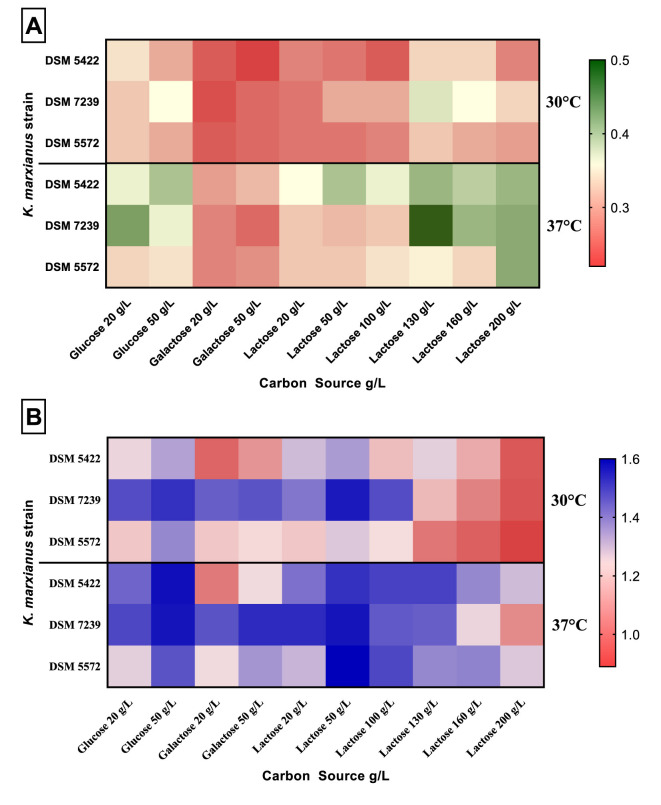
Specific maximum growth rate (μ_max_) **(A)** and maximum OD_600_ **(B)** on different carbon sources (glucose, galactose, lactose) at different concentrations (glucose and galactose at 20 and 50 g/L; lactose 20–200 g/L), and temperatures (30 and 37 °C) for *K. marxianus* DSM 5422, DSM 7239, and DSM 5572 in MTP growth experiments.

Figure 1 reports μ_max_ and maximum OD_600_ on the three different carbon sources at various concentrations (glucose and galactose at 20 and 50 g/L; lactose 20–200 g/L) and temperatures (30 and 37 °C) for the three *K. marxianus* strains.

The yeasts exhibited different growth patterns in μ_max_ (Figure 1A) and biomass production (Figure 1B). Both the values of μ_max_ and OD_600_ were higher at 37 °C compared to those obtained at 30 °C.

Moreover, μ_max_ increased as a function of the increase in lactose concentration. It reached the highest value at 130 g/L lactose (Figure 1A; Table 2). A further increase in lactose concentration up to 200 g/L negatively affected the μ_max_ of all the strains. Silveira et al. (2005) observed the same trend by growing *K. marxianus* at lactose concentration higher than 130 g/L at the flask scale. This trend was observed for all the strains at both 30 and 37 °C, with the sole exception of the strain DSM 5572 grown at 37 °C, which obtained the highest μ_max_ value on lactose 200 g/L.

*K. marxianus* DSM 7329 reached the highest μ_max_ value of 0.50 h^−1^ with respect to all the MTP growth tests, as shown by the dark green square cell in the heatmap (Figure 1A).

The OD_600_ values were characterized by an inverse pattern: the biomass production decreased as a function of the increase in lactose concentration (Figure 1B; Table 2). Specifically, for all the yeasts, it reached the highest values at 50 g/L lactose at both temperatures, whereas the lowest optical densities were obtained in the presence of 200 g/L lactose in the SMM.

### 3.2 β-galactosidase tests

The majority of biotechnological processes currently developed to utilize whey's sugar content are based on the enzymatic hydrolysis of lactose in whey by β-galactosidase (Kaur et al., 2009; Bilal et al., 2022). Since the efficient conversion of lactose into glucose and galactose is an essential step in lactose exploitation, the β-galactosidase activity of the selected strains was investigated to evaluate possible differences between them (Varela et al., 2017). Since lactose induces the expression of β-galactosidases, cultures were grown in a lactose-containing medium, according to the literature (Liu et al., 2022). Based on the results previously obtained from the MTP growth test (Section 3.1), especially in terms of maximum OD_600_ values, the SSM-50 g/L lactose was selected as culture medium for the β-galactosidase tests.

In order to better understand if there was an influence of the different phases of cell growth on enzyme activity, the β-galactosidase test was conducted after 6 h (during the exponential phase) and 16 h (during the stationary phase), respectively (Bacci Junior et al., 1996), as reported in Table 3. Moreover, the activity tests were also carried out on permeabilized and non-permeabilized cells. The reported results showed the mean values of three independent experiments ± the standard deviation. Values within the same column that show different letters are significantly different (*p* < 0.05).

**Table 3:** Specific β-galactosidase activity expressed as U/mg and U/mL (for the supernatant) of *K. marxianus* cells growth in SSM-50 g/L lactose at 30 °C.

***K. marxianus* strain**	**Specific activity (**μ**mol mL**^**−1**^ **min**^**−1**^ **mg**^**−1**^**)**			
	**Permeabilized cells (6 h)**	**Permeabilized cells (16 h)**	**Non-permeabilized cells (16 h)**	**Supernatant of non-permeabilized cells (16 h)** ^*^
DSM 5422	20.85 ± 0.74^c^	22.08 ± 1.73^b^	0.41 ± 0.06^a^	2.89 ± 0.26^a^
DSM 7239	27.20 ± 1.82^a^	27.80 ± 0.90^a^	0.96 ± 0.07^a^	3.24 ± 0.27^a^
DSM 5572	23.77 ± 0.98^b^	24.86 ± 1.28^c^	0.59 ± 0.03^a^	2.89 ± 0.41^a^

Specific activity was determined during the exponential phase (6 h) and in the early stationary phase (16 h) of permeabilized cells, in the stationary phase of non-permeabilized cells (16 h) and the supernatant of non-permeabilized cells (16 h).

^*^Values were expressed as μmol·10^−3^ mL^−1^ min^−1^. Values within the same column that show different letters are significantly different (*p* < 0.05).

**Figure 2:**
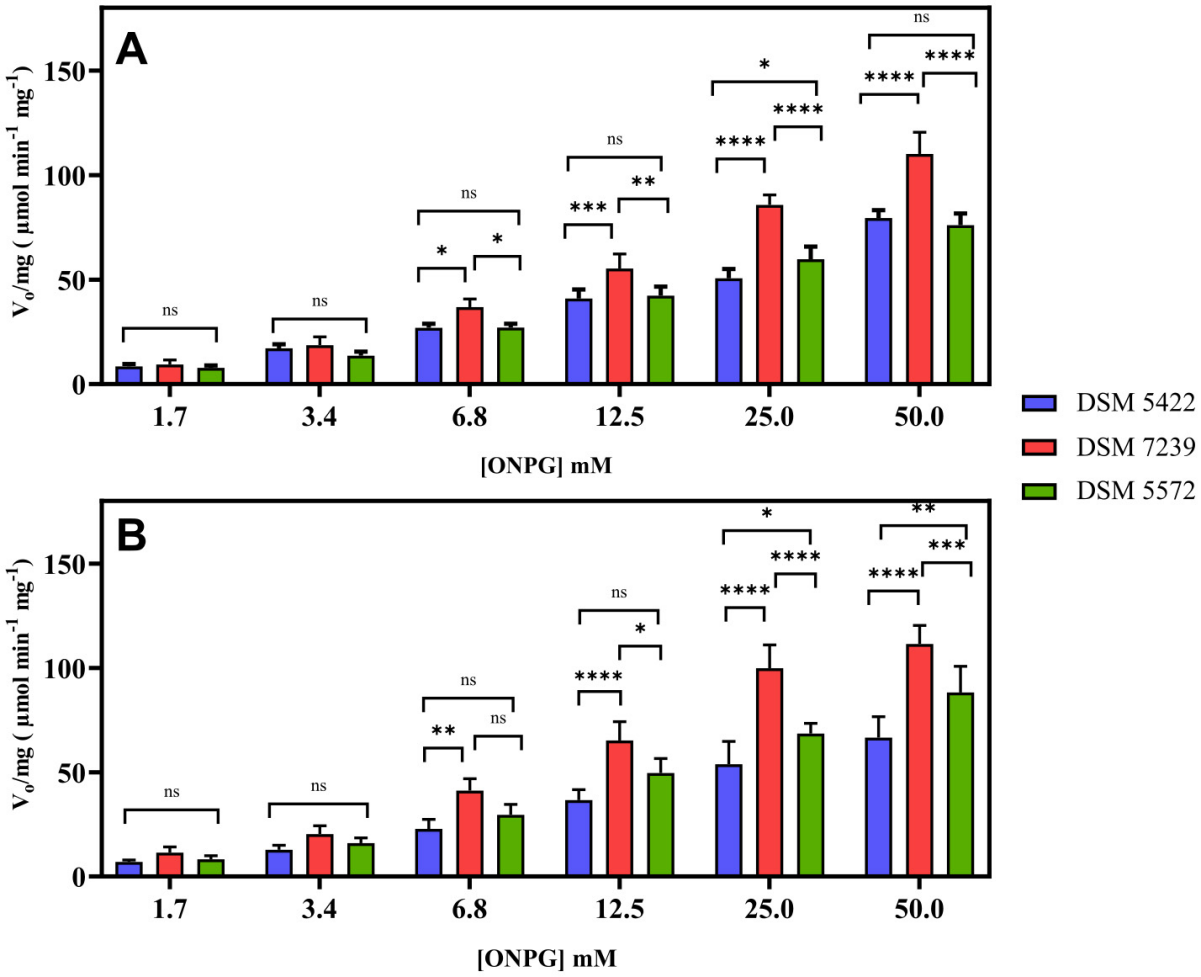
Effect of the ONPG concentration (1.7–50 mM) on the β-galactosidase activity of each permeabilized *K. marxianus* strain, expressed as V_0_ (μmol min^−1^) per mg of biomass, at 30 °C **(A)** and 37 °C **(B)**. Statistical significance was established at *p* < 0.05 and marked by *p < 0.05, **p < 0.01, ***p < 0.001, and ****p < 0.0001.

Kinetic studies were conducted in the presence of various ONPG concentrations in order to evaluate the enzymatic performance of *K. marxianus* at 30 and 37 °C. The results obtained were reported in Figure 2. They show the mean value of at least three independent experiments together with the standard deviation.

The initial reaction rates (V_0_) were determined by measuring the slope of the linear portion of the product concentration vs. the time plot, and it was expressed as V_0_ (μmol min^−1^) per mg of biomass. This approach enabled a direct comparison of the efficiency across different temperatures and substrate concentrations. Statistical results from the ANOVA analysis on the measured V_0_/mg showed significant differences in the class “Temperature” and “[ONPG]” with a *p*-*value* < 0.0001, whereas multiple comparisons between each condition couple showed no significant difference between the two temperatures.

As shown in Figure 2A, there was no statistical difference between the strains at lower concentrations of ONPG at 30 °C. However, at higher substrate concentrations, the V_0_ of the strain DSM 7239 was clearly greater than that of strains DSM 5422 and DSM 5572. The maximum initial rate (V_0_/mg) was reached for higher concentrations of ONPG (25 and 50 mM), with marked significance of the difference between DSM 7239 and the other two strains, while no statistically significant differences were found between the strains DSM 5422 and DSM 5572.

The pattern of an increased reaction rate in relation to increasing ONPG concentrations was also confirmed at 37 °C (Figure 2B). Notably, a significant difference was observed between the three strains, with DSM 7239 appearing to be the most efficient at higher ONPG concentrations (between 12.5 and 50 mM) compared with the other two strains studied. However, a two-way ANOVA statistical analysis of the results obtained at the two temperatures and among the three strains did not reveal any statistical difference or improvement between the two tested conditions.

Relevant kinetic parameters, such as K_M_ and V_max_, of the hydrolysis reaction of ONPG to ONP catalyzed by permeabilized cells were calculated and listed in Table 4. Results showed the mean values of three independent experiments ± the standard deviations. Statistical analyses were performed using a multiple two-tailed Student's *t*-test. Statistical significance was determined using the Holm-Sidak method, with α = 0.05. Values within the same column that share the same letters are not significantly different.

**Table 4:** Kinetic parameters, namely K_M_ (mM) and V_max_ (μmol min^−1^ mg^−1^), calculated for ONPG hydrolysis performed by permeabilized *K. marxianus* strains at 30 and 37 °C.

***K. marxianus* strain**	**K**_**M**_ **(mM)**		**V**_**max**_ **(**μ**mol min**^**−1**^ **mg**^**−1**^**)**	
	**30** °**C**	**37** °**C**	**30** °**C**	**37** °**C**
DSM 5422	23.27 ± 2.06^a^	25.06 ± 7.11^a^	90.69 ± 18.12^a^	95.87 ± 30.03^a^
DSM 7239	31.99 ± 9.60^a^	50.09 ± 16.98^bc^	224.03 ± 53.29^b^	285.45 ± 110.91^b^
DSM 5572	27.93 ± 3.86^a^	31.05 ± 2.99^ac^	124.92 ± 12.85^a^	157.26 ± 23.76^c^

Values within the same column that show different letters are significantly different (*p* < 0.05).

### 3.3 Flask fermentation tests

To assess the difference in lactose utilization efficiency and relative ethanol production at various temperatures (30, 37, and 42 °C), a flask-scale up was conducted on three *K. marxianus* strains using SSM-lactose at 50 g/L. The improved results obtained at 37 °C (see Section 3.1) and the well-known thermotolerance of *K. marxianus* led to the decision to increase the temperature further, up to 42 °C.

**Figure 3:**
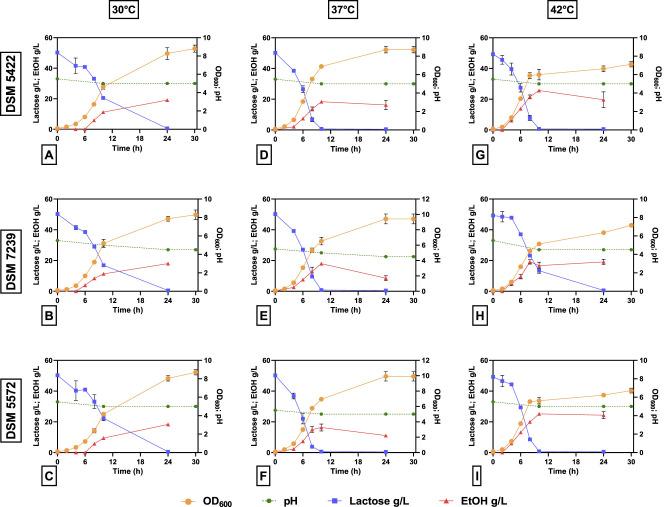
Growth profile (OD_600_), pH, lactose consumption, and ethanol production in 40 mL of SSM-50 g/L lactose liquid medium at different temperatures (30, 37, and 42 °C) for *K. marxianus* DSM 5422 **(A, D, G)**, *K. marxianus* DSM 7239 **(B, E, H)**, and *K. marxianus* DSM 5572 **(C, F, I)**. The right y-axis is referred to OD_600_ (orange circles) and pH (green circles); the left y-axis is referred to lactose (blue squares) and ethanol (red triangles) concentrations (g/L).

Figure 3 shows the kinetics of lactose conversion, ethanol production, biomass concentration (OD_600_) and pH variation. Values represent the mean ± standard deviation of three independent experiments.

In all cases, after 24 h, the complete consumption of lactose was observed. In particular, at 37 and 42 °C, the complete consumption of lactose occurred within 10 h, confirming a positive effect on the yeast metabolism of this sugar. In contrast, about half of the initial carbon source remained after 10 h at 30 °C for all strains. Moreover, *K. marxianus* DSM 7239 consumed lactose at a slower rate during the first 8 h, even though no discernible differences were seen between the three strains at 10 h. The amount of ethanol produced by the three strains (~20 g/L) was not significantly different, but the maximum concentration was reached at different times depending on the strain and temperature. Similarly, biomass production (~8–9 OD_600_) was similar for all three yeast strains at each temperature. A slight decrease in biomass production was observed for each strain by moving from 30 to 42 °C. Furthermore, the cells entered the stationary phase after 10 h of fermentation at 42 °C (Figures 3G–I), whereas the stationary phase was reached after 24 h for all the strains at 30 and 37 °C.

Finally, the pH remained almost constant throughout the fermentation process in all cases. From an industrial point of view, this is a crucial advantage since it avoids the need for strict pH control during the process and reduces operational costs.

Table 5 reports the main fermentation outputs, in terms of μ_max_, dry biomass production (g/L) after 1 day of the process, lactose consumption after 10 h, ethanol production after 10 and 24 h, yield of the product with respect to the substrate (Y_P/S_) and yield of biomass with respect to the substrate (Y_X/S_) after 24 h of fermentation. The results shown are the mean values of three independent experiments.

**Table 5:** Main process outputs of shake flask fermentation tests of *K. marxianus* DSM 5422, *K. marxianus* DSM 7239, and *K. marxianus* DSM 5572 at 30, 37, and 42 °C in 40 mL of SSM liquid medium supplemented with 50 g/L of lactose.

***K. marxianus* strain**	**Temperature (°C)**	**μ_max_ (h^−1^)**	**Biomass (g/L)^a^**	**Consumed lactose (g/L)^b^**	**EtOH (g/L) 10 h**	**EtOH (g/L) 24 h**	**Y_P/S_ (g/g)^c^**	**Y_X/S_ (g/g)^d^**
DSM 5422	30	0.36	5.50	26.40	11.23	19.15^*^	0.38 ± 0.04	0.11
	37	0.46	5.77	49.42	18.38	16.27	0.37 ± 0.03	0.11
	42	0.59	4.41	48.63	25.68	19.66^*^	0.53 ± 0.02	0.09
DSM 7239	30	0.41	5.23	30.54	11.32	18.05^*^	0.36 ± 0.03	0.10
	37	0.48	5.41	49.29	17.99	8.53	0.36 ± 0.02	0.11
	42	0.57	4.22	35.94	16.66	19.11	0.39 ± 0.1	0.09
DSM 5572	30	0.37	5.35	24.29	9.43^*^	18.30	0.36 ± 0.04	0.11
	37	0.45	5.71	49.49	16.32	11.06	0.33 ± 0.11	0.11
	42	0.57	4.14	48.54	25.15	24.33	0.52 ± 0.02	0.08

^*^Values with RSD > 10% (for all the other values, RSD was < 10%).

^a^Values referred to dry biomass concentration after 24 h of fermentation.

^b^Values referred to 10 h of the process.

^c^Y_P/S_ was calculated when the substrate was completely consumed; confidence intervals are reported, calculated at 95% confidence level (n = 3).

^d^Y_X/S_ was calculated after 24 h of fermentation.

According to previous MTP tests, an increase in temperature positively impacted μ_max_ for all strains, even at flask scale. Furthermore, increasing the temperature to 42 °C further improved the μ_max_ values, reaching approximately 0.6 h^−1^. However, biomass production did not follow the same trend. The highest biomass concentrations were achieved for all three strains at 37 °C, which corresponded with the complete consumption of lactose (around 49.5 g/L) after 10 h.

Regarding ethanol production, an increase in temperature favored its production for all yeasts, despite a decrease in biomass growth. *K. marxianus* DSM 5422 and DSM 5572 performed best, achieving maximum ethanol titers of 25.7 and 25.2 g/L, respectively, after 10 h of fermentation at 42 °C.

The ethanol yield on the substrate (Y_P/S_) did not differ significantly among the yeast strains at 30 and 37 °C but increased significantly at 42 °C for *K. marxianus* DSM 5422 and DSM 5572, reaching values of 0.53 ± 0.02 g/g and 0.52 ± 0.02 g/g, respectively. These values were close to the maximum theoretical ethanol yield. Conversely, biomass yield values on the substrate (Y_X/S_) did not differ significantly among strains or at different temperatures, remaining within the range of 0.08–0.11 g/g.

### 3.4 Fermentation tests in lactose-rich medium

**Figure 4:**
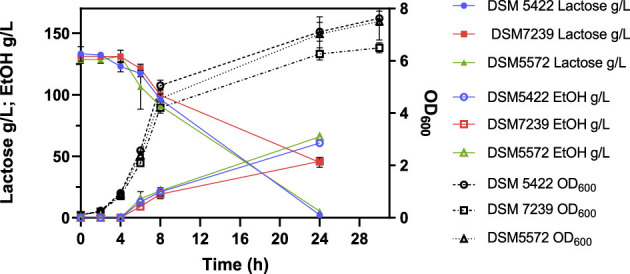
Fermentation profile of *K. marxianus* DSM 5422, DSM 7239, and DSM 5572 at shake-flask scale carried out at 42 °C in 40 mL of SSM-130 g/L lactose liquid medium.

To validate the MTP screening test findings further, the performance of the yeast strains was evaluated at the optimal fermentation temperature of 42 °C and lactose concentration of 130 g/L in the SSM. These conditions were chosen based on the highest observed specific growth rate during MTP screening, in order to confirm whether the previously identified optimal temperature and lactose concentration consistently yielded superior fermentation performance in a flask setup. Figure 4 shows the kinetic profiles of lactose conversion, ethanol production, and cell growth (OD_600_) over the process time. Values are the mean ± standard deviation of three independent experiments.

Table 6 reports the main fermentation outputs, in terms of μ_max_, dry biomass production (g/L), lactose consumption, ethanol production, yield of the product with respect to the substrate (Y_P/S_), and yield of biomass with respect to the substrate (Y_X/S_). All these parameters were calculated after 24 h of fermentation.

**Table 6:** Main process outputs of shake flask fermentation tests of *K. marxianus* DSM 5422, *K. marxianus* DSM 7239, and *K. marxianus* DSM 5572 at 42 °C in 40 mL of SSM liquid medium supplemented with 130 g/L of lactose after 24 h.

***K. marxianus* strain**	**μ_max_ (h^−1^)**	**Biomass (g/L)**	**Consumed lactose (g/L)**	**EtOH (g/L)**	**Y_P/S_ (g/g)^a^**	**Y_X/S_ (g/g)**
DSM 5422	0.50	6.06	127.52	60.74	0.48 ± 0.03	0.05
DSM 7239	0.48	4.36	84.77	41.61	0.49 ± 0.03	0.05
DSM 5572	0.49	6.00	124.44	62.52	0.50 ± 0.03	0.05

Results were the mean values of three independent experiments, and RSD values were < 15%.

^a^For Y_P/S_ (g/g) values, confidence intervals are reported, calculated at 95% confidence level (n = 3).

All the strains showed comparable μ_max_ values, which were reproducible with respect to those observed in the shake flask tests on SMM-50 g/L lactose (Table 5). Strains DSM 5422 and DSM 5572 were characterized by the highest DCW concentration of 6 g/L, while *K. marxianus* DSM 7239 produced around 4.4 g/L of biomass. This last value was similar to that achieved by the same strain in the presence of 50 g/L lactose (Table 5). Moreover, DSM 5422 and DSM 5572 showed almost complete lactose consumption, producing 60.7 g/L and 62.5 g/L of ethanol, respectively, corresponding to yields of 0.48 ± 0.03 g/g and 0.50 ± 0.03 g/g, respectively (Table 6). In contrast, strain DSM 7239 consumed only 65.3% of the initial lactose amount after 24 h, producing a lower concentration of ethanol, equal to 41.6 g/L, corresponding to a yield of 0.49 ± 0.03 g/g. This value was similar to those achieved for the other two strains.

### 3.5 Fermentation of cheese whey permeate to bioethanol

**Figure 5:**
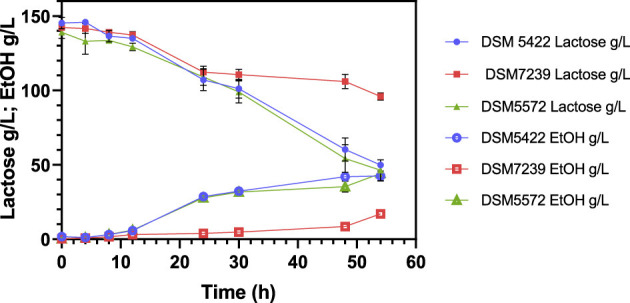
Fermentation profile of *K. marxianus* DSM 5422, DSM 7239, and DSM 5572 at shake-flask scale at 42 °C in 40 mL of CWP medium, containing around 140 g/L lactose, without any other supplementation.

The main innovative aspect of this study was the sustainable and efficient exploitation of CWP (a strategic and impactful agro-industrial waste) via lactose fermentation to produce bioethanol rapidly and efficiently. The potential of *K. marxianus* strains for ethanol production was then investigated using a lactose-rich CWP medium containing approximately 140 g/L of lactose and no additional supplements, at a temperature of 42 °C. Figure 5 shows the kinetic profiles of lactose conversion and ethanol production over time. Values represent the mean ± standard deviation of three independent experiments.

In line with previous findings on lactose-rich synthetic media (Figure 4), *K. marxianus* DSM 5422 and DSM 5572 exhibited the greatest lactose consumption efficiency in the CWP, despite their slower consumption rate compared to that observed in the SSM-130 g/L lactose medium (Figure 4). Specifically, lactose was completely metabolized in the SSM after 24 h, while around 65% of the carbon source was consumed in the CWP medium in 54 h (Figure 5). The pH remained stable throughout the entire process (data not shown), indicating that although the process was slowed down, and its stability was unaffected. Furthermore, the growth of *K. marxianus* DSM 7239 was significantly inhibited by the CWP. This strain was only able to convert around 30% of the initial lactose, resulting in a significantly lower dry cell weight and ethanol concentration compared to the other two strains, as shown in Table 7.

**Table 7:** Main process outputs of shake flask fermentation tests of *K. marxianus* DSM 5422, *K. marxianus* DSM 7239, and *K. marxianus* DSM 5572 at 42 °C in 40 mL of CWP medium (around 140 g/L lactose) without any other supplementation.

***K. marxianus* strain**	**Biomass (g/L)**	**Consumed lactose (g/L)**	**EtOH (g/L)**	**Y_P/S_ (g/g)^a^**	**Y_X/S_ (g/g)**
DSM 5422	5.51	95.37	42.44	0.50 ± 0.13	0.06
DSM 7239	3.18	46.27	17.03	0.37 ± 0.09	0.07
DSM 5572	5.75	89.19	44.17	0.50 ± 0.04	0.06

Results were the mean values of three independent experiments, and RSD values were < 10%.

^a^For Y_P/S_ (g/g) values, confidence intervals are reported, calculated at 95% confidence level (n = 3).

Table 7 reports the main fermentation outputs, in terms of dry biomass production (g/L), lactose consumption, ethanol production, yield of the product with respect to the substrate (Y_P/S_), and yield of biomass with respect to the substrate (Y_X/S_). All these parameters were calculated after 54 h of fermentation. Due to the turbid nature of the CWP substrate, which is rich in particulate matter, it was difficult to record the OD data required to construct a growth curve and calculate μ_max_ values. For this reason, DCW concentration was only used to measure biomass production.

*K. marxianus* DSM 5422 and DSM 5572 produced around 5.5 and 5.8 g/L of dry biomass, respectively (Table 7). These values were slightly lower than those (~6 g/L) reached by the same strains in the SSM (Table 6). In contrast, *K. marxianus* DSM 7239 exhibited lower cell growth of approximately 3.2 g/L after 54 h of fermentation (Table 7). In this case, the DCW concentration was significantly lower than that achieved in SSM fermentation (~4.4 g/L) (Table 6). Similar trends were observed for lactose conversion: *K. marxianus* DSM 5422 and DSM 5572 consumed around 90 g/L, whereas *K. marxianus* DSM 7239 converted half of this amount.

Strains DSM 5422 and DSM 5572 produced a comparable amount of ethanol (around 43 g/L), while DSM 7239 exhibited significantly lower ethanol production (only 17 g/L).

Although ethanol production in CWP was lower than in SSM, the Y_P/S_ and Y_X/S_ values were very similar in the two processes (Tables 6, 7). The maximum Y_P/S_ values were achieved by the DSM 5422 and DSM 5572 strains, which were close to the maximum theoretical yield.

## 4 Discussion

Considering the high specific growth rate of *K. marxianus* DSM 5422 in the presence of a wide range of lactose concentrations (20–200 g/L), it can be considered a promising candidate for industrial applications in modern biorefinery schemes. Moreover, in the preliminary screening, the temperature of 37 °C represented the most favorable condition for the growth of the selected yeast strain. The optimal lactose concentration for the maximization of μ_max_ was 130 g/L for both *K. marxianus* DSM 5422 and DSM 7239. Lactose concentration in CWP is usually comprised in the range 140–180 g/L (Díez-Antolínez et al., 2016). For this reason, the preliminary results obtained for the three strains in MTP tests encouraged further scale-up to the flask to validate these findings. Similar results on the physiology of *K. marxianus* were reported by Saini et al. (2017), who found that an increase in sugar concentration in the fermentation medium up to 150 g/L of lactose led to a corresponding rise in colony-forming units of these yeast strains. However, higher concentrations generated significant osmotic stress for cells, which caused a decrease in cell vitality and the quantified colony-forming units (Saini et al., 2017).

Regarding the β-galactosidase activity test, statistically significant differences were not observed in the activity between the exponential growth phase and the stationary phase of the selected yeasts. These findings were consistent with previous studies (Bacci Junior et al., 1996). On the contrary, significant differences in enzymatic activity were observed between the yeast strains. In particular, *K. marxianus* DSM 7239 showed a maximum β-galactosidase activity of 27.8 ± 0.9 U mg^−1^ after 16 h of incubation.

As reported in the study of Rollini et al. (2008), in most *K. marxianus* strains, β-galactosidase is primarily located inside the cell (as intracellular enzyme). Preliminary tests conducted on the supernatant of non-permeabilized cells confirmed the intracellular localization for all the strains studied in the present work. Moreover, tests conducted on untreated cells, as a control, indicated that permeabilization of cells increased the β-galactosidase activity, which is mainly located inside the cells. Not significant differences were detected between the yeast strains under the adopted test conditions. These findings are in accordance with other similar studies (Bacci Junior et al., 1996; Rollini et al., 2008).

Regarding the kinetic study of β-galactosidase activity through the monitoring of the ONPG hydrolysis to give ONP, the K_M_ values of the three strains and their enzymes' affinity for the substrate were similar at 30 °C and slightly different at 37 °C. Differently, significant differences were observed in V_max_ values, both at 30 and 37 °C. The significantly higher V_max_ for the DSM 7239 strain could indicate that the catalytic activity of its β-galactosidase is intrinsically higher in the adopted experimental conditions. Moreover, this result could also be related to the overexpression of β-galactosidase by this strain, namely, a higher amount of enzyme produced per unit of biomass, compared to the other strains. At the same time, the absence of any significant differences in the enzymatic activity of β-galactosidase between the exponential and stationary growth phases excluded a diverse level of expression of the enzyme in different cell-cycle stages. These results aligned with the higher β-galactosidase activity (V_0_/mg) observed at increasing ONPG concentrations at both temperatures considered.

The K_M_ values obtained for *K. marxianus* DSM 5422, DSM 7239, and DSM 5572 in the present investigation (23–50 mM) were higher than those reported in the literature for other strains, such as *K. marxianus* ATCC 16045 (2.3 mM) and *K. marxianus* CCT 7082 (3.3 mM) (Cavalcante Braga et al., 2013; Selvarajan and Mohanasrinivasan, 2015). This difference may be due to the use of a crude enzyme preparation from permeabilized cells in this study, as opposed to other studies where the test was conducted using purified enzymes (Osorio-González et al., 2022). Furthermore, the permeabilization process can increase the usefulness of CW for yeast that does not consume lactose. For example, permeabilized *K. marxianus* cells were used in co-culture with *S. cerevisiae* in hydrolysed CW (Yadav et al., 2014) to produce mixed-culture biomass. Another example of its application was the use of permeabilized *K. marxianus* cells for lactose hydrolysis in CW. This was then fermented by *Aureobasidium pullulans* in a mixed culture to produce polymalic acid. This expands the applications of *K. marxianus* to co-culture systems for other fermentative processes involving non-lactose-consuming microorganisms (Xia et al., 2021).

The enzyme kinetics study revealed substantial inter-strain variability in β-galactosidase performance, despite the similarity in substrate affinity (K_M_). Across both tested temperatures (30 °C and 37 °C), the three strains exhibited comparable K_M_ values, suggesting that the affinity of their β-galactosidases for the synthetic substrate ONPG is not the major determinant of their physiological differences. However, clear divergence emerged in their maximum catalytic rate (V_max_) under high substrate availability.

Among the strains, DSM 7239 consistently displayed the highest V_max_ values, especially at elevated ONPG concentrations. These kinetic features, correlate well with the high specific activities measured in permeabilized cell extracts (27.8 ± 0.9 U mg^−1^), reinforcing the notion that DSM 7239 is the most efficient lactose-hydrolyser.

By contrast, DSM 5422 and DSM 5572 showed lower V_max_ values, reflecting a reduced enzymatic activity compared with DSM 7239. However, these strains exhibited similar kinetic profiles to each other, with no significant differences in hydrolytic activity across the range of ONPG concentrations tested. This suggests that their enzymatic machinery for lactose hydrolysis is more balanced, leading to steadier lactose assimilation during fermentation.

Regarding flask fermentation on SSM-50 g/L lactose, μ_max_ values were higher at flask scale than in MTP tests. Nevertheless, the micro-cultivation experimental set-up proved useful for predicting the physiological behavior of the investigated strains. Indeed, at both the MTP and flask scales, a temperature of 37 °C produced a better specific growth rate than 30 °C. Metabolic activity in yeast produces heat, causing the temperature of the bioreactor to rise during fermentation. Using thermotolerant yeast strains could therefore play a significant role in high-temperature fermentation, as it reduces the risk of contamination and cooling costs at an industrial scale (Saini et al., 2017). Another attractive feature is that *K. marxianus* showed a higher specific growth rate at 42 °C, which gives it an advantage over bacteria in a non-sterile industrial process (Pendón et al., 2021). Most bacterial and fungal contaminants of industrial fermentations thrive below 40 °C; thus, maintaining the process at ≥42 °C provides a selective environment favoring thermotolerant yeasts such as *K. marxianus* (Pendón et al., 2021). This reduces the need for costly sterilization steps, which represent a significant burden in large-scale operations (Tesfaw, 2023). Lower contamination pressure translates into greater process robustness, reduced downtime, and lower operating costs in non-sterile biorefineries (Saini et al., 2017). Another benefit relates to energy balance. Fermentation processes generate metabolic heat, and cooling is a substantial cost factor in large bioreactors. By using strains capable of performing efficiently at 42–45 °C, the cooling demand is reduced (Pattanakittivorakul et al., 2024). This aligns with the goal of lowering the carbon footprint of bioprocessing. However, running at higher temperatures has implications for yeast physiology and medium composition. *K. marxianus* is known to tolerate up to 52 °C (Fonseca et al., 2008), but performance differences between strains are significant, as shown in this study. Above 42 °C, certain strains exhibit reduced growth or impaired lactose uptake, which may limit ethanol productivity if not carefully matched with process design (Kosaka et al., 2022). Additionally, prolonged exposure to elevated temperatures can increase cellular stress, potentially leading to reduced viability in repeated-batch or continuous fermentations (Fu et al., 2019). This may necessitate strain adaptation, evolutionary engineering, or process strategies such as cell recycling to maintain stable performance. Medium composition also becomes critical at high temperature (Flores-Cosío et al., 2024). In complex nutrient-poor substrates, such as CWP, nitrogen limitation may interact synergistically with thermal stress, slowing down lactose metabolism. Supplementation with low-cost nitrogen sources (e.g., urea or corn steep liquor) may therefore be required in large-scale applications to sustain fermentation rates under non-sterile, thermophilic conditions. Finally, downstream processing considerations should not be overlooked. Higher fermentation temperatures can facilitate ethanol recovery by distillation, as the feed enters distillation columns closer to operating conditions, thereby reducing energy inputs (Kumakiri et al., 2021). However, foaming and viscosity issues may be exacerbated under thermophilic conditions and must be managed through process engineering.

For all these reasons, 42 °C was selected as the optimal temperature for this process and was investigated in shake flask experiments using SSM-lactose-rich and CWP-based media.

To validate the fermentation results obtained in the preliminary MTP tests in the presence of a higher lactose concentration (130 g/L), similar to that in the CWP, a flask-scale investigation of the physiological performance of the three strains was performed at 42 °C. DSM 7239 displayed the highest β-galactosidase specific activity, as well as superior catalytic efficiency at elevated ONPG concentrations compared to DSM 5422 and DSM 5572. However, despite this enhanced hydrolytic capacity, DSM 7239 consumed lactose more slowly, accumulated less biomass, and produced significantly less ethanol in both semi-synthetic media and CWP. This apparent contradiction highlights that efficient lactose hydrolysis does not necessarily translate into efficient fermentation. Several factors may contribute to this outcome. First, although DSM 7239 is proficient in breaking down lactose into glucose and galactose, its ability to channel these monosaccharides into central carbon metabolism and alcoholic fermentation pathways may be impaired at high substrate concentrations and elevated temperatures (Fu et al., 2019; Kosaka et al., 2022). Indeed, the strain showed slower lactose utilization kinetics at 42 °C compared to DSM 5422 and DSM 5572, suggesting that heat stress may negatively affect downstream metabolic steps such as galactose catabolism or glycolytic flux regulation, even if hydrolysis itself is not limiting (Beniwal et al., 2017). In addition, excessive galactose release could contribute to a metabolic bottleneck, since galactose assimilation in yeasts is typically slower than glucose and is tightly regulated (Beniwal et al., 2017). If galactose metabolism is inefficient, this could lead to suboptimal carbon flux into ethanol production, despite efficient lactose cleavage (Lyutova and Naumova, 2023). Third, the nutrient composition of CWP may exacerbate this imbalance. With its low nitrogen and micronutrient content, CWP imposes additional stress on yeast metabolism. DSM 7239, which already shows sensitivity to elevated temperature and osmotic pressure, may be more vulnerable to these combined stresses, further reducing its fermentative performance. In contrast, DSM 5422 and DSM 5572, while showing lower β-galactosidase activity, appear to balance lactose hydrolysis with efficient assimilation of glucose and galactose, achieving near-theoretical ethanol yields even in nutrient-limited whey permeate. Taken together, these findings suggest that DSM 7239 is an effective lactose utilizer rather than an efficient ethanol producer. Its high enzymatic activity could make it an attractive candidate for biotechnological applications where lactose hydrolysis itself is the desired outcome (e.g., production of lactose-free dairy products or bioconversion processes requiring glucose and galactose release). However, for ethanol production from CWP, DSM 5422 and DSM 5572 represent superior candidates due to their balanced metabolism, thermotolerance, and robustness in nutrient-limited conditions.

Díez-Antolínez et al. (2016) conducted flask fermentation tests in synthetic media supplemented with 130 g/L lactose at 35 °C for 48 h, using strains DSM 5422 and DSM 7239. They reported ethanol production of 52.9 and 48.8 g/L for these strains, respectively, corresponding to yields of 0.41 and 0.36 g/g. In the present study, a higher ethanol yield was observed for DSM 5422 (0.48 ± 0.03 g/g with production of around 60 g/L of ethanol) in just 24 h. In contrast, DSM 7239 exhibited lower ethanol production (41.61 g/L) than that reported by Dìez-Antolìnez et al., but a higher yield (0.49 ± 0.03 g/g). As the compositions of the media used in this study and in the study by Dìez-Antolìnez et al. were similar, it can be concluded that the impaired growth and slower lactose consumption rate observed in DSM 7239 were due to its sensitivity to higher temperatures (42 °C instead of 35 °C).

This physiological behavior, observed in the fermentation of CWP by all strains with respect to SSM, could be explained by the low concentration of amino acids (an organic nitrogen source) in CWP. While a high C/N ratio generally supports fermentation (Sezmis et al., 2018; Pendón et al., 2021), the presence of sufficient nitrogen in the medium is crucial for efficient alcoholic fermentation, and deficiency can negatively impact overall fermentation performance (Prazeres et al., 2012).

As reported in Section 2.3, the CWP provided by the Italian company Distilleria Bartin Srl contained only 1 g/L of total nitrogen and around 140 g/L of lactose. This corresponds to a very high C/N ratio in the presence of a relatively low concentration of the nitrogen source.

Furthermore, although higher temperatures offer several advantages, such as a higher lactose consumption rate, as observed in the previous experiment with SSM and 130 g/L of lactose, the combined effects of high-temperature stress and deficiency in nitrogen and other micronutrients likely impaired lactose consumption in the test. These conditions may have enhanced the inhibitory effect of other factors, such as osmotic stress (Tesfaw, 2023).

Industrial fermentation introduces a variety of stressors that can impact the performance and viability of microbial strains (Pattanakittivorakul et al., 2024; Rocha et al., 2011; Saini et al., 2017). In CWP fermentation processes, these stressors include high temperatures, osmotic stress, organic acids, and ethanol accumulation (Rocha et al., 2011; Tesfaw, 2023). Thus, determining the “safe” range of temperature, substrate, product and inhibitor concentrations is essential in this context (Dreżek et al., 2023). While *K. marxianus* is known for its thermotolerance (Fonseca et al., 2008), high temperatures can still pose challenges by destabilizing cellular structures and inhibiting metabolic processes: proteins and membranes are particularly susceptible to thermal damage, which can lead to reduced growth rates and lower ethanol yields (Fu et al., 2019; Montini et al., 2022). Since low lactose content results in low ethanol titer, direct whey to bioethanol fermentation is not cost-effective (Tesfaw, 2023). Consequently, starting the fermentation with higher lactose concentrations is one of the key strategies to achieve a higher ethanol concentration (Saini et al., 2017). However, a high amount of sugar causes osmotic stress that results in reduced growth and cell survival, which prevents most yeast strains from growing and fermenting high-gravity substrate (Díez-Antolínez et al., 2016; Saini et al., 2017). CWP also contains salts, mainly NaCl, KCl, and calcium phosphates (Prazeres et al., 2012). Salts, together with the high lactose concentration, contribute to osmotic stress affecting membrane integrity, impairing nutrient transport, and increasing the energy demand for maintaining cellular homeostasis. Wongso reported that ethanol fermentation by *K. marxianus* is not affected by NaCl concentrations up to 35 g/L, suggesting a degree of salt tolerance that varies by strain and environmental conditions (Wongso, 1993). The by-product of acid coagulation is acid whey. Either organic acids like citric or acetic are added during its processing. In particular, acid whey is a by-product of making Greek yogurt and acid-coagulated cheeses, such as ricotta and cottage cheese (Rocha-Mendoza et al., 2021). These weak acids can inhibit cell growth and fermentation performance (Martynova et al., 2016). Ethanol, the desired product of CWP fermentation, can act as an inhibitor at high concentrations by disrupting membrane fluidity and impairing metabolic processes (Saini et al., 2017). Ethanol stress can also exacerbate the effects of other stressors, such as osmotic pressure and temperature (Costa et al., 2014; Saini et al., 2017).

Moreover, regarding the toxic effect of high concentrations of ethanol on *K. marxianus*, in the present study, strains DSM 5422 and DSM 5572 showed cells vitality up to 61–63 g/L on SSM liquid medium supplemented with 130 g/L of lactose after 24 h, demonstrating a high resistance to high ethanol concentration. Our results are consistent with what was observed by Costa et al. (2014), who studied the *K. marxianus* strains ATCC 8554 and CCT 4086 able to withstand ethanol concentrations as high as 60 g/L, while the *K. marxianus* UFV-3 strain was only able to grow at concentrations as high as 40 g/L. The ability of *K. marxianus* to tolerate ethanol is a critical trait for its application in industrial bioethanol production. Several studies have shown that the typical ethanol tolerance of *K. marxianus* strains is around 60 g/L (Costa et al., 2014; Diniz et al., 2017). Dreżek et al. (2023) investigated the effect of exogenous ethanol on the growth of *K. marxianus* WUT240. Ethanol was added when cultures reached an OD_600_ of 0.8–1.0, at final concentrations of 12.5–100 g/L, alongside a control without ethanol. Growth, monitored via OD_600_ measurements, showed a 47% reduction compared to the control at ethanol concentrations of 62.5 g/L, while concentrations of 75 and 100 g/L almost completely inhibited growth (77% and 88%, respectively). However, through adaptive evolution or metabolic engineering, this tolerance could be significantly enhanced (Fu et al., 2019). For instance, da Silveira et al. reported that initial ethanol tolerance in *K. marxianus* strains could be improved from 60 to 100 g/L following an adaptive laboratory evolution (ALE) strategy. This improvement was attributed to metabolic changes, including increased accumulation of ergosterol and unsaturated fatty acids (UFAs), which stabilized the cell membrane, both in control and stress conditions (da Silveira et al., 2020). Furthermore, Pal and Vij (2022) found that the ALE approach increased the ethanol tolerance of *K. marxianus* MTCC1389 strain from 80 to 120 g/L, leading to a 42.9% improvement in ethanol production (110.5 g/L vs. 78.3 g/L). Similarly, Mo et al. (2019) demonstrated that evolved *K. marxianus* strains subjected to ethanol stress over 100 days developed enhanced tolerance through transcriptional regulation of multiple metabolic pathways, such as sterol biosynthesis and membrane fortification, suggesting that in *K. marxianus*, ethanol tolerance improvement perhaps relied primarily on transcriptional regulation rather than DNA mutation.

Based on the results of the present study, *K. marxianus* DSM 5422 was found to be the most suitable microorganism for converting CWP into bioethanol, thanks to its high biomass production, lactose conversion and ethanol yield. Using *K. marxianus* DSM 5422 in a modern biorefinery scheme to valorize CWP is a strategic move, as it eliminates the need to purchase commercial β-galactosidase enzymes at an industrial scale, thanks to its natural enzymatic activity. This makes the process more cost-effective and scalable. Furthermore, its ability to grow on a wide range of substrates and lactose concentrations (50–200 g/L), with high specific growth rates at temperatures of up to 42 °C, ensures high ethanol yields (very close to the maximum theoretical value), making this GRAS yeast strain a strategic microorganism for CWP and CW valorization in the context of the circular bioeconomy.

In conclusion, these results are important, especially from the perspective of the process scalability and economic feasibility of the proposed biorefinery scheme, as reported in our recent study (Colacicco et al., 2024) focused on the process scale-up simulation and techno-economic assessment of ethanol fermentation from cheese whey using *K. marxianus*. The process was simulated for a facility in Apulia, Italy, treating 539 m^3^/day of CW. The model integrated three steps: membrane filtration to recover proteins and concentrate lactose, fermentation to ethanol, and anaerobic digestion of residues for energy and soil conditioner. Three scenarios with different yields and lactose concentrations were assessed. Techno-economic analysis showed ethanol production costs ranging from 1.43 to 2.57 *e*/kg, with the best performance achieved at higher substrate concentrations and improved yeast efficiency. Sensitivity analysis revealed strong dependence on CW cost or credit, highlighting the importance of policy frameworks. Overall, CW ethanol production is feasible, especially under a gate-fee system, and could serve as a sustainable plug-in solution for dairy-based biorefineries.

## 5 Conclusions

This study investigated three promising commercial strains of the yeast species *Kluyveromyces marxianus* (DSM 5422, KDSM 7239, and DSM 5572) as attractive candidates for the sustainable production of bioethanol from CWP at high lactose concentration and high temperatures. Preliminary physiological characterization of the *K. marxianus* strains was performed using MTP tests on synthetic media containing various lactose concentrations (50–200 g/L) at 30 and 37 °C. The natural activity of the β-galactosidase enzyme in the different strains was studied alongside a kinetic study in an MTP assay to correlate fermentation performance with β-galactosidase expression. Subsequently, a flask-scale study was conducted in an SSM containing 130 g/L lactose at temperatures of 30, 37, and 42 °C to evaluate the impact of temperature on yeast growth, lactose conversion, and ethanol production. Optimal conditions for lactose utilization and ethanol production were identified as 130 g/L lactose at 42 °C for all yeasts. However, *K. marxianus* DSM 5422 exhibited superior performance in both synthetic media and real CWP liquid medium by achieving high lactose conversion (approximately 65% of the initial concentration, equivalent to around 95 g/L), substantial biomass (approximately 6 g/L) and significant ethanol production (approximately 43 g/L), resulting in an ethanol yield of 0.50 ± 0.13 g/g. Notably, this value is very close to the maximum theoretical limit. These findings emphasized the importance of selecting appropriate fermentation conditions to maximize yield while maintaining process stability. By expanding knowledge of *K. marxianus* physiology and optimizing its performance in CWP fermentation, this research has contributed to the development of economically and environmentally sustainable bioethanol production processes, while addressing the valorization of a major agro-industrial waste product from the dairy sector.

## Data Availability

The original contributions presented in the study are included in the article/supplementary material, further inquiries can be directed to the corresponding author.

## References

[B1] Arshad U. T. Hassan A. Ahmad T. Naeem M. Chaudhary M. T. Abbas S. Q.. (2023). A recent glance on the valorisation of cheese whey for industrial prerogative: high-value-added products development and integrated reutilising strategies. Int. J. Food Sci. Technol. 58, 2001–2013. 10.1111/ijfs.16168

[B2] Asunis F. De Gioannis G. Dessì P. Isipato M. Lens P.N.L. Muntoni A.. (2020). The dairy biorefinery: integrating treatment processes for cheese whey valorisation. J. Environ. Manag. 276:111240. 10.1016/j.jenvman.2020.111240 32866754

[B3] Awasthi M. K. Paul A. Kumar V. Sar T. Kumar D. Sarsaiya S.. (2022). Recent trends and developments on integrated biochemical conversion process for valorisation of dairy waste to value added bioproducts: a review. Bioresour. Technol. 344:126193. 10.1016/j.biortech.2021.126193 34710613

[B4] Bacci Junior M. Siqueira C. G. Antoniazi S. A. Ueta J. (1996). Location of the β-galactosidase of the yeast *Kluyveromyces marxianus* var. *marxianus* ATCC 10022. Anton. Leeuw. 69, 357–361. 10.1007/BF00399624 8836433

[B5] Bansal S. Oberoi H. S. Dhillon G. S. Patil R. T. (2008). Production of β-galactosidase by *Kluyveromyces marxianus* MTCC 1388 using whey and effect of four different methods of enzyme extraction on β-galactosidase activity. Indian J. Microbiol. 48, 337–341. 10.1007/s12088-008-0019-0 23100731 PMC3476777

[B6] Barba F. (2021). An integrated approach for the valorisation of cheese whey. Foods 10:564. 10.3390/foods10030564 33803106 PMC8002121

[B7] Becerra M. Cerdan E. Gonzalez Siso M.I. (1998). Dealing with different methods for Kluyveromyces lactis β-galactosidase purification. Biol. Proced. Online 1, 48–58. 10.1251/bpo4 12734592 PMC140120

[B8] Beniwal A. Saini P. Kokkiligadda A. Vij S. (2017). Physiological growth and galactose utilisation by dairy yeast *Kluyveromyces marxianus* in mixed sugars and whey during fermentation. 3 Biotech 7:349. 10.1007/s13205-017-0985-1 28955646 PMC5612877

[B9] Bilal M. Ji L. Xu Y. Xu S. Lin Y. Iqbal H. M. N.. (2022). Bioprospecting *Kluyveromyces marxianus* as a robust host for industrial biotechnology. Front. Bioeng. Biotechnol. 10:851768. 10.3389/fbioe.2022.851768 35519613 PMC9065261

[B10] Bradford M.M. (1976). A rapid and sensitive method for the quantitation of microgram quantities of protein utilizing the principle of protein-dye binding. Anal. Biochem. 72, 248–254. 10.1016/0003-2697(76)90527-3 942051

[B11] Caballero A. Caballero P. León F. Rodríguez-Morgado B. Martín L. Parrado J.. (2021). Conversion of whey into value-added products through fermentation and membrane fractionation. Water 13:1623. 10.3390/w13121623

[B12] Carvalho P. Costa C. E. Baptista S. I .L. Domingues L. (2021). Yeast cell factories for sustainable whey-to-ethanol valorisation towards a circular economy. Biofuel Res. J. 10.18331/BRJ2021.8.4.4

[B13] Cavalcante Braga A. R. Manera A. P. da Costa Ores J. Sala L. Maugeri F. Juliano Kalil S. (2013). Kinetics and thermal properties of crude and purified β-galactosidase with potential for the production of galactooligosaccharides. Food Technol. Biotechnol. 51, 45–52.

[B14] Colacicco M. De Micco C. Macrelli S. Agrimi G. Janssen M. Bettiga M.. (2024). Process scale-up simulation and techno-economic assessment of ethanol fermentation from cheese whey. Biotechnol. Biofuels 17:124. 10.1186/s13068-024-02567-5 39342290 PMC11439329

[B15] Costa D. A. De Souza C. J. A. Costa P. S. Rodrigues M. Q. R. B. Dos Santos A. F. Lopes M. R.. (2014). Physiological characterization of thermotolerant yeast for cellulosic ethanol production. Appl. Microbiol. Biotechnol. 98, 3829–3840. 10.1007/s00253-014-5580-3 24535257 PMC3973951

[B16] da Silveira F. A. de Oliveira Soares D. L. Bang K. W. Balbino T. R. de Moura Ferreira M. A. Diniz R. H. S.. (2020). Assessment of ethanol tolerance of Kluyveromyces marxianus CCT 7735 selected by adaptive laboratory evolution. Appl. Microbiol. Biotechnol. 104, 7483–7494. 10.1007/s00253-020-10768-9 32676708

[B17] de Albuquerque T. L. de Sousa M. Gomes e Silva N.C. Girão Neto C.A.C. Barros Gonçalves L.R. Fernandez-Lafuente R.. (2021). β-Galactosidase from *Kluyveromyces lactis*: characterization, production, immobilization and applications-A review. Int. J. Biol. Macromol. 191, 881–898. 10.1016/j.ijbiomac.2021.09.133 34571129

[B18] de Faria J. T. Rocha P. F. Converti A. Passos F. M. L. Minim L. A. Sampaio F. C. (2013). Statistical investigation of *Kluyveromyces lactis* cells permeabilization with ethanol by response surface methodology. Braz. J. Microbiol. 44, 1067–1074. 10.1590/S1517-83822013000400007 24688494 PMC3958170

[B19] Díez-Antolínez R. Hijosa-Valsero M. Paniagua A.I. Gómez X. (2016). Very-high-gravity fermentation of non-supplemented cheese whey permeate by immobilized *Kluyveromyces marxianus*. Chem. Eng. Trans. 49, 529–534. 10.3303/CET1649089

[B20] Díez-Antolínez R. Hijosa-Valsero M. Paniagua-García A.I. Garita-Cambronero J. Gómez X. (2018). Yeast screening and cell immobilization on inert supports for ethanol production from cheese whey permeate with high lactose loads. PLoS One 13:e0210002. 10.1371/journal.pone.0210002 30596755 PMC6312371

[B21] Diniz R. H. S. Villada J. C. Alvim M. C. T. Vidigal P. M. P. Vieira N. M. Lamas-Maceiras M.. (2017). Transcriptome analysis of the thermotolerant yeast Kluyveromyces marxianus CCT 7735 under ethanol stress. Appl. Microbiol. Biotechnol. 101, 6969–6980. 10.1007/s00253-017-8432-0 28776098

[B22] Dinkci N. (2021). Whey, waste or value? World J. Agric. Soil Sci. 648, 1–5. 10.33552/WJASS.2021.06.000648

[B23] Dreżek K. Sobczyk M. K. Kállai Z. Detman A. Bardadyn P. Mierzejewska J. (2023). Valorisation of whey permeate in sequential bioprocesses towards value-added products–optimisation of biphasic and classical batch cultures of *Kluyveromyces marxianus*. Int. J. Mol. Sci. 24:7560. 10.3390/ijms24087560 37108722 PMC10146618

[B24] El-Tanboly E.S. El-Hofi M. K. Khorshid A. (2017). Recovery of cheese whey, a by-product from the dairy industry for use as an animal feed. J. Nutri. Health Food Eng. 6:148. 10.15406/jnhfe.2017.06.00215

[B25] Figueroa Pires A. Marnotes N. G. Rubio O. D. Garcia A. C. Pereira C. D. (2021). Dairy by-products: a review on the valorisation of whey and second cheese whey. Foods 10:1067. 10.3390/foods10051067 34066033 PMC8151190

[B26] Flores-Cosío G. García-Béjar J. A. Sandoval-Nuñez D. Amaya-Delgado L. (2024). Stress response and adaptation mechanisms in *Kluyveromyces marxianus*. Adv. Appl. Microbiol. 126, 27–62. 10.1016/bs.aambs.2024.02.003 38637106

[B27] Fonseca G.G. Heinzle E. Wittmann C. Gombert A.K. (2008). The yeast *Kluyveromyces marxianus* and its biotechnological potential. Appl. Microbiol. Biotechnol. 79, 339–354. 10.1007/s00253-008-1458-6 18427804

[B28] Fonseca G. G. Barbosa de Carvalho N. M. Gombert A. K. (2013). Growth of the yeast *Kluyveromyces marxianus* CBS 6556 on different sugar combinations as sole carbon and energy source. Appl. Microbiol. Biotechnol. 97, 5055–5067. 10.1007/s00253-013-4748-6 23435899

[B29] Fu X. Li P. Zhang L. Li S. (2019). Understanding the stress responses of *Kluyveromyces marxianus* after an arrest during high-temperature ethanol fermentation based on integration of RNA-Seq and metabolite data. Appl. Microbiol. Biotechnol. 103, 2715–2729. 10.1007/s00253-019-09637-x 30673809

[B30] Griffith K. L. Wolf Jr. R. E. (2002). Measuring β-galactosidase activity in bacteria: cell growth, permeabilization, and enzyme assays in 96-well arrays. Biochem. Biophys. Res. Commun. 290, 397–402. 10.1006/bbrc.2001.6152 11779182

[B31] Inchaurrondo V. A. Yantorno O. M. Voget C. E. (1994). Yeast growth and β-galactosidase production during aerobic batch cultures in lactose-limited synthetic medium. Process Biochem. 29, 47–54. 10.1016/0032-9592(94)80058-8

[B32] Karim A. Gerliani N. Aïder M. (2020). *Kluyveromyces marxianus*: an emerging yeast cell factory for applications in food and biotechnology. Int. J. Food Microbiol. 333:108818. 10.1016/j.ijfoodmicro.2020.108818 32805574

[B33] Kaur G. Panesar P.S. Bera M.B. Kumar H. (2009). Hydrolysis of whey lactose using CTAB-permeabilized yeast cells. Bioprocess Biosyst. Eng. 32, 63–67. 10.1007/s00449-008-0221-9 18431601

[B34] Kosaka T. Tsuzuno T. Nishida S. Pattanakittivorakul S. Murata M. Miyakawa I.. (2022). Distinct metabolic flow in response to temperature in thermotolerant *Kluyveromyces marxianus*. Appl. Environ. Microbiol. 88:e02006–21. 10.1128/aem.02006-21 35080905 PMC8939337

[B35] Kumakiri I. Yokota M. Tanaka R. Shimada Y. Kiatkittipong W. Lim J. W.. (2021). Process intensification in bio-ethanol production–recent developments in membrane separation. Processes 9:1028. 10.3390/pr9061028

[B36] Lane M. M. Burke N. Karreman R. Wolfe K. H. O'Byrne C. P. Morrissey J. P. (2011). Physiological and metabolic diversity in the yeast *Kluyveromyces marxianus*. Anton. Leeuw. 100, 507–519. 10.1007/s10482-011-9606-x 21674230

[B37] Lappa I. K. Papadaki A. Kachrimanidou V. Terpou A. Koulougliotis D. Eriotou E. (2019). Cheese whey processing: integrated biorefinery concepts and emerging food applications. Foods 8:347. 10.3390/foods8080347 31443236 PMC6723228

[B38] Leandro M. J. Marques S. Ribeiro B. Santos H. Fonseca C. (2019). Integrated process for bioenergy production and water recycling in the dairy industry: selection of *Kluyveromyces* strains for direct conversion of concentrated lactose-rich streams into bioethanol. Microorganisms 7:545. 10.3390/microorganisms7110545 31717512 PMC6920800

[B39] Liu B. Wu P. Zhou J. Yin A. Yu Y. Lu H. (2022). Characterization and optimization of the LAC4 upstream region for low-leakage expression in *Kluyveromyces marxianus*. Yeast 39, 283–296. 10.1002/yea.3682 34791694

[B40] Löser C. Urit T. Gruner E. Bley T. (2015). Efficient growth of *Kluyveromyces marxianus* biomass used as a biocatalyst in the sustainable production of ethyl acetate. Energ. Sustain. Soc. 5, 1–15. 10.1186/s13705-014-0028-2

[B41] Lyutova L. V. Naumova E. S. (2023). Comparative analysis of fermentation of lactose and its components, glucose and galactose, by interstrain hybrids of dairy yeast *Kluyveromyces lactis*. Appl. Biochem. Microbiol. 59, 1150–1156. 10.1134/S0003683823090119

[B42] Martynova J. Kokina A. Kibilds J. Liepins J. Scerbaka R. Vigants A. (2016). Effects of acetate on *Kluyveromyces marxianus* DSM 5422 growth and metabolism. Appl. Microbiol. Biotechnol. 100, 4585–4594. 10.1007/s00253-016-7392-0 26910042

[B43] Mo W. Wang M. Zhan R. Yu Y. He Y. Lu H. (2019). *Kluyveromyces marxianus* developing ethanol tolerance during adaptive evolution with significant improvements of multiple pathways. Biotechnol. Biofuels 12, 1–15. 10.1186/s13068-019-1393-z 30949239 PMC6429784

[B44] Montini N. Doughty T. W. Domenzain I. Fenton D. A. Baranov P. V. Harrington R.. (2022). Identification of a novel gene required for competitive growth at high temperature in the thermotolerant yeast *Kluyveromyces marxianus*. Microbiology 168:1148. 10.1099/mic.0.001148 35333706 PMC9558357

[B45] Morrissey J. P. Etschmann M. M. W. Schrader J. de Billerbeck G. M. (2015). Cell factory applications of the yeast *Kluyveromyces marxianus* for the biotechnological production of natural flavour and fragrance molecules. Yeast 32, 3–16. 10.1002/yea.3054 25393382

[B46] Nobre Macedo A. T. Z. Oliveira Monteiro J. F. da Silva Azedo D. J. C. Duarte E. Pereira C. D. (2020). “Membrane applications for lactose recovering” in Lactose and Lactose Derivatives, ed. *N. Gutiérrez-Mendéz* (London: IntechOpen).

[B47] Ortiz-Merino R. A. Varela J. A. Coughlan A. Y. Hoshida H. Da Silveira W. B. Wilde C. (2018). Ploidy variation in *Kluyveromyces marxianus* separates dairy and non-dairy isolates. Front. Genet. 9:94. 10.3389/fgene.2018.00094 29619042 PMC5871668

[B48] Osorio-González C. S. Gómez-Falcon N. Brar S. K. Ramírez A. A. (2022). Cheese whey as a potential feedstock for producing renewable biofuels: a review. Energies 15:6828. 10.3390/en15186828

[B49] Ozmihci S. Kargi F. (2007a). Comparison of yeast strains for batch ethanol fermentation of cheese–whey powder (CWP) solution. Lett. Appl. Microbiol. 44, 602–606. 10.1111/j.1472-765X.2007.02132.x 17576220

[B50] Ozmihci S. Kargi F. (2007b). Continuous ethanol fermentation of cheese whey powder solution: effects of hydraulic residence time. Bioprocess Biosyst. Eng. 30, 79–86. 10.1007/s00449-006-0101-0 17143639

[B51] Ozmihci S. Kargi F. (2007c). Effects of feed sugar concentration on continuous ethanol fermentation of cheese whey powder solution (CWP). Enzyme Microb. Technol. 41, 876–880. 10.1016/j.enzmictec.2007.07.01517143639

[B52] Ozmihci S. Kargi F. (2007d). Kinetics of batch ethanol fermentation of cheese-whey powder (CWP) solution as function of substrate and yeast concentrations. Bioresour. Technol. 98, 2978–2984. 10.1016/j.biortech.2006.10.005 17118651

[B53] Pal U. Vij S. (2022). Adaptive evolution of Kluyveromyces marxianus MTCC1389 for high ethanol tolerance. Biocatal. Agric. Biotechnol. 45:102533. 10.1016/j.bcab.2022.102533

[B54] Palmieri N. Forleo M. B. Salimei E. (2017). Environmental impacts of a dairy cheese chain including whey feeding: an Italian case study. J. Clean. Prod. 140, 881–889. 10.1016/j.jclepro.2016.06.185

[B55] Panesar P. S. Panesar R. Singh R. S. Kennedy J. F. Kumar H. (2006). Microbial production, immobilization and applications of β-D-galactosidase. J. Chem. Technol. Biotechnol. 81, 530–543. 10.1002/jctb.1453

[B56] Pasotti L. Zucca S. Casanova M. Micoli G. Cusella De Angelis M. G. Magni P. (2017). Fermentation of lactose to ethanol in cheese whey permeate and concentrated permeate by engineered *Escherichia coli*. BMC Biotechnol. 17, 1–12. 10.1186/s12896-017-0369-y 28577554 PMC5457738

[B57] Pattanakittivorakul S. Kumakiri I. Nutaratat P. Hara M. Yokota M. Murata M.. (2024). High-temperature fermentation and its downstream processes for compact-scale bioethanol production. Fuels 5, 857–867. 10.3390/fuels5040048

[B58] Pendón M. D. Madeira J. V. Romanin D. E. Rumbo M. Gombert A. K. Garrote G. L. (2021). A biorefinery concept for the production of fuel ethanol, probiotic yeast, and whey protein from a by-product of the cheese industry. Appl. Microbiol. Biotechnol. 105, 3859–3871. 10.1007/s00253-021-11278-y 33860834

[B59] Peri K. V. R. Yuan L. Faria Oliveira F. Persson K. Alalam H. D. Olsson L. (2024). A unique metabolic gene cluster regulates lactose and galactose metabolism in the yeast *Candida intermedia*. Appl. Environ. Microbiol. 90:e01135–01124. 10.1128/aem.01135-24 39240082 PMC11497787

[B60] Prazeres A. R. Carvalho F. Rivas J. (2012). Cheese whey management: a review. J. Environ. Manag. 110, 48–68. 10.1016/j.jenvman.2012.05.018 22721610

[B61] Rocha J. M. Guerra A. (2020). On the valorisation of lactose and its derivatives from cheese whey as a dairy industry by-product: an overview. Eur. Food Res. Technol. 246, 2161–2174. 10.1007/s00217-020-03580-2

[B62] Rocha S. N. Abrahão-Neto J. Gombert A. K. (2011). Physiological diversity within the *Kluyveromyces marxianus* species. Anton. Leeuw. 100, 619–630. 10.1007/s10482-011-9617-7 21732033

[B63] Rocha-Mendoza D. Kosmerl E. Krentz A. Zhang L. Badiger S. Miyagusuku-Cruzado G.. (2021). Acid whey trends and health benefits. J. Dairy Sci. 104, 1262–1275. 10.3168/jds.2020-19038 33358165

[B64] Rollini M. Trinetta V. Musatti A. Manzoni M. (2008). Influence of substrate on β-galactosidase production by *Kluyveromyces* strains. Ann. Microbiol. 58, 705–710. 10.1007/BF03175578

[B65] Rouanet A. Bolca S. Bru A. Claes I. Cvejic H. Girgis H. (2020). Live biotherapeutic products, a road map for safety assessment. Front. Med. 7:237. 10.3389/fmed.2020.00237 32637416 PMC7319051

[B66] Rubio-Texeira M. (2006). Endless versatility in the biotechnological applications of *Kluyveromyces* LAC genes. Biotechnol. Adv. 24, 212–225. 10.1016/j.biotechadv.2005.10.001 16289464

[B67] Saini P. Beniwal A. Kokkiligadda A. Vij S. (2017). Evolutionary adaptation of *Kluyveromyces marxianus* strain for efficient conversion of whey lactose to bioethanol. Process Biochem. 62, 69–79. 10.1016/j.procbio.2017.07.013

[B68] Selvarajan E. Mohanasrinivasan V. (2015). Kinetic studies on exploring lactose hydrolysis potential of β-galactosidase extracted from *Lactobacillus plantarum* HF571129. J. Food Sci. Technol. 52, 6206–6217. 10.1007/s13197-015-1729-z 26396367 PMC4573140

[B69] Sezmis A. L. Malerba M. E. Marshall D. J. McDonald M. J. (2018). Beneficial mutations from evolution experiments increase rates of growth and fermentation. J. Mol. Evol. 86, 111–117. 10.1007/s00239-018-9829-9 29349600

[B70] Sheetz R. M. Dickson R. C. (1980). Mutations affecting synthesis of β-galactosidase activity in the yeast *Kluyveromyces lactis*. Genetics 95, 877–890. 10.1093/genetics/95.4.877 6781984 PMC1214274

[B71] Silveira W. B. Passos F. J. V. Mantovani H. C. Passos F. M. L. (2005). Ethanol production from cheese whey permeate by *Kluyveromyces marxianus* UFV-3: a flux analysis of oxido-reductive metabolism as a function of lactose concentration and oxygen levels. Enzyme Microb. Technol. 36, 930–936. 10.1016/j.enzmictec.2005.01.018

[B72] Singh R.V. Sambyal K. (2023). β-Galactosidase as an industrial enzyme: production and potential. Chem. Pap. 77, 11–31. 10.1007/s11696-022-02507-3

[B73] Tesfaw A. (2023). The current trends of bioethanol production from cheese whey using yeasts: biological and economical perspectives. Front. Energy Res. 11:1183035. 10.3389/fenrg.2023.1183035

[B74] Tsalis T. A. Malamateniou K. E. Koulouriotis D. Nikolaou I. E. (2020). New challenges for corporate sustainability reporting: United Nations' 2030 Agenda for sustainable development and the sustainable development goals. Corp. Soc. Responsib. Environ. Manag. 27, 1617–1629. 10.1002/csr.1910

[B75] Varela J. A. Montini N. Scully D. Van der Ploeg R. Oreb M. Boles E.. (2017). Polymorphisms in the LAC12 gene explain lactose utilisation variability in *Kluyveromyces marxianus* strains. FEMS Yeast Res. 17:fox021. 10.1093/femsyr/fox021 28444380

[B76] Wongso D. D. (1993). Optimisation of Industrial Whey Ethanol Fermentation Process. Palmerston North: Massey University.

[B77] Xia J. He J. Xu J. Liu X. Qiu Z. Xu N.. (2021). Direct conversion of cheese whey to polymalic acid by mixed culture of *Aureobasidium pullulans* and permeabilized *Kluyveromyces marxianus*. Bioresour. Technol. 337:125443. 10.1016/j.biortech.2021.125443 34171705

[B78] Yadav J. S. S. Bezawada J. Ajila C. M. Yan S. Tyagi R. D. Surampalli R. Y. (2014). Mixed culture of *Kluyveromyces marxianus* and *Candida krusei* for single-cell protein production and organic load removal from whey. Bioresour. Technol. 164, 119–127. 10.1016/j.biortech.2014.04.069 24844166

[B79] Zandona E. BlaŽić M. ReŽek Jambrak A. (2021). Whey utilisation: sustainable uses and environmental approach. Food Technol. Biotechnol. 59, 147–161. 10.17113/ftb.59.02.21.6968 34316276 PMC8284110

[B80] Zotta T. Solieri L. Iacumin L. Picozzi C. Gullo M. (2020). Valorisation of cheese whey using microbial fermentations. Appl. Microbiol. Biotechnol. 104, 2749–2764. 10.1007/s00253-020-10408-2 32009200

[B81] Zou J. Chang X. (2022). Past, present, and future perspectives on whey as a promising feedstock for bioethanol production by yeast. J. Fungi 8:395. 10.3390/jof8040395 35448626 PMC9031875

[B82] Zou J. Guo X. Shen T. Dong J. Zhang C. Xiao D. (2013). Construction of lactose-consuming *Saccharomyces cerevisiae* for lactose fermentation into ethanol fuel. J. Ind. Microbiol. Biotechnol. 40, 353–363. 10.1007/s10295-012-1227-5 23344501

